# Dynamics of Dynamin during Clathrin Mediated Endocytosis in PC12 Cells

**DOI:** 10.1371/journal.pone.0002416

**Published:** 2008-06-11

**Authors:** Joshua Z. Rappoport, Katherine P. Heyman, Shahrnaz Kemal, Sanford M. Simon

**Affiliations:** Laboratory of Cellular Biophysics, The Rockefeller University, New York, New York, United States of America; Thomas Jefferson University, Kimmel Cancer Center, United States of America

## Abstract

**Background:**

Members of the dynamin super-family of GTPases are involved in disparate cellular pathways. Dynamin1 and dynamin2 have been implicated in clathrin-mediated endocytosis. While some models suggest that dynamin functions specifically at the point of vesicle fission, evidence also exists for a role prior to fission during vesicle formation and it is unknown if there is a role for dynamin after vesicle fission. Although dynamin2 is ubiquitously expressed, dynamin1 is restricted to the nervous system. These two structurally similar endocytic accessory proteins have not been studied in cells that endogenously express both.

**Methodology/Principal Findings:**

The present study quantitatively assesses the dynamics of dynamin1 and dynamin2 during clathrin-mediated endocytosis in PC12 cells, which endogenously express both proteins. Both dynamin isoforms co-localized with clathrin and showed sharp increases in fluorescence intensity immediately prior to internalization of the nascent clathrin-coated vesicle. The fluorescence intensity of both proteins then decreased with two time constants. The slower time constant closely matched the time constant for the decrease of clathrin intensity and likely represents vesicle movement away from the membrane. The faster rate may reflect release of dynamin at the neck of nascent vesicle following GTP hydrolysis.

**Conclusions/Significance:**

This study analyses the role of dynamin in clathrin-mediated endocytosis in a model for cellular neuroscience and these results may provide direct evidence for the existence of two populations of dynamin associated with nascent clathrin-coated vesicles.

## Introduction

Three *bona fide* dynamin genes have been described, and dynamin gene products exist in a large number of splice variants with potentially distinct expression patterns and functions [1]–[3]. Both dynamin1 and dynamin2 are thought to be involved in clathrin-mediated endocytosis [1]. Additionally, dynamins and dynamin related proteins have been implicated in such varied cellular processes as organelle biogenesis (e.g. mitochondria), membrane ruffling, actin regulation, nitric oxide production, and clathrin-independent endocytosis [4]–[9]. The observation of aberrant long-necked clathrin-coated vesicles at the neuron-muscular junction in the drosophila *shibire* dynamin mutant, as well as in cells treated with GTPγS, suggested a role in fission of nascent vesicles from the cell surface, potentially as a mechano-enzyme [10], [11]. Subsequently, it was also suggested that dynamin could be involved in formation of clathrin-coated vesicles, possibly as a regulatory GTPase [12]–[15].

While the expression of dynamin1 is limited to cells of the nervous system, dynamin2 exists in a ubiquitous expression pattern [2]. The role of dynamin in endocytosis has been evaluated through genetic, biochemical, biophysical, and ultra-structural studies [16]. More recently, several studies have attempted to glean functional information from live-cell imaging of dynamin at sites of endocytosis [17]–[22]. Each study of dynamin1 has revealed an increase of fluorescence just before disappearance of clathrin spots from the cell surface, the moment of endocytosis [19], [20], [23], [24]. Clathrin intensity was relatively constant prior to the burst of dynamin fluorescence. Thus, these results could be interpreted as increased recruitment of dynamin to the necks of fully formed nascent clathrin-coated vesicles immediately prior to the fission event. Following the peak of dynamin fluorescence a very rapid decrease was observed. Although this could reflect the loss of dynamin self-association upon GTP hydrolysis, such phenomena have yet to be quantitatively analyzed.

Of the studies imaging dynamin2 during clathrin-mediated endocytosis, some have suggested that both dynamin2 and clathrin exhibit a gradual rise in fluorescence intensity, potentially more consistent with function as a regulatory GTPase [22], [25]. However, others have demonstrated dynamics more similar to that of dynamin1 [18], [26]. Interestingly, each study imaging dynamin2 during clathrin-mediated endocytosis has been performed with a different cell line, further suggesting the potential importance of cell type in studies of protein function. An additional caveat to these results is that none of these studies was performed in cells that endogenously express dynamin1. This point is of concern as several known dynamin1-interacting proteins demonstrate restriction distributions of expression as well [27], [28]. Thus, the present study was designed to quantitatively compare the dynamics of dynamin1 and dynamin2 directly against each other during clathrin-mediated endocytosis in a cell line known to express both isoforms.

PC12 cells, derived from a pheochromocytoma of the rat adrenal medulla, are a dynamin1 positive neuroendocrine cell line that has been used to image exocytosis by TIR-FM [29]–[33]. Although PC12 cells have previously been used in some studies of dynamin, clathrin-mediated endocytosis has not been analyzed by live-cell imaging in these cells [34]. The results of our analysis demonstrate that, as in other cell lines, both dynamin1 and dynamin2 co-localize significantly with clathrin at the cell surface. Furthermore, our evaluation of the dynamics of dynamin1 and clathrin in PC12 cells demonstrate findings similar to those observed in other cell lines that do not endogenously express dynamin1. Strikingly, in PC12 cells the dynamics of dynamin2 were nearly identical to those of dynamin1.

Immediately following the rise in fluorescence, dynamin intensity dropped with two time constants. One population of dynamin (70%) decreased in fluorescence at the same rate as clathrin or internalized cargo, consistent with being the result of movement of the vesicle out of the excitatory evanescent field. A second population (30%) decreased much faster (20–40 fold) and may represent dynamin dissociated from the vesicle. Similar results were observed for dynamin1 and dynamin2. Thus, imaging individual events of endocytosis provides evidence for two dynamin populations associated with clathrin-mediated endocytosis: one that is rapidly released as well as one that internalizes associated with the nascent clathrin-coated vesicle.

## Materials and Methods

### Plasmid constructs:

All constructs were the same as we employed in previous studies [35]. The construct encoding rat clathrin light chain A tagged with dsRed (clathrin-dsRed) was a gift from Tomas Kirchhausen (Harvard Medical School, Boston, MA). Human dynamin1aa tagged with EGFP (pEGFPn1-dynamin1aa) was a gift from Pietro de Camilli (Yale University, New Haven, CT). Rat dynamin2aa tagged with EGFP (pEGFPn1-dynamin2aa) was a gift from Mark McNiven (The Mayo Clinic, Rochester, MI).

### Cell Culture:

PC12 cells were obtained from American Type Culture Collection (Manassas, VA) and were cultured in DMEM (Invitrogen, Carlsbad, CA) supplemented with 10% horse serum and 5% fetal bovine serum in a humidified 37°C incubator with 5% CO_2_. Cells were plated onto MatTek 35 mm glass bottom dishes (MatTek Corporation, Ashland, MA) coated with Mouse Collagen Type IV (BD Biosciences, San Jose, CA), according to the supplier's directions. Cells were transfected using Lipofectamine 2000 (Invitrogen, Carlsbad, CA) according to the supplier's directions. All imaging was performed 48 hours post-transfection.

### TIR-FM image acquisition:

TIR-FM was performed at 37°C using an Apo 60× 1.45 NA microscope objective (Olympus America, Melville, NY), as previously described [36]. Clathrin-dsRed and EGFP-labeled dynamin1 or dynamin2 were excited with the 488-nm line of a tunable argon laser (Omnichrome, model 543-AP A01, Melles Griot, Carlsbad, CA) reflected off a 498-nm dichroic mirror. All mirrors and filters were obtained from Chroma Technologies (Brattleboro, VT). Green and red emissions were collected simultaneously using a dual-view splitter (Optical Insights, Santa Fe, NM) equipped with a 515/30-nm band-pass filter to collect green emission, a 550-nm dichroic mirror to split the emission, and a 580-nm long-pass filter to collect red emission. The camera utilized to acquire images was a ORCA-ER (Hamamatsu Photonics, Bridgewater, NJ). The camera and a mechanical shutter (Uniblitz, Vincent Associates, Rochester, NY) were controlled by MetaMorph (Universal Imaging, Downingtown, PA). Images were acquired utilizing 300 ms exposures times, and 100 frames were streamed to memory on a PC during acquisition and then saved to hard disk.

### Dual-color processing:

After subtraction of extracellular background, 12-bit dual-color TIR-FM image streams were aligned in MetaMorph. On the basis of single-fluorophore control experiments, green-to-red bleed-through corrections of 10% for were employed; following background subtraction and alignment, 10% of the EGFP signal was subtracted from dsRed images.

### Data analysis:

**Figure 1:**
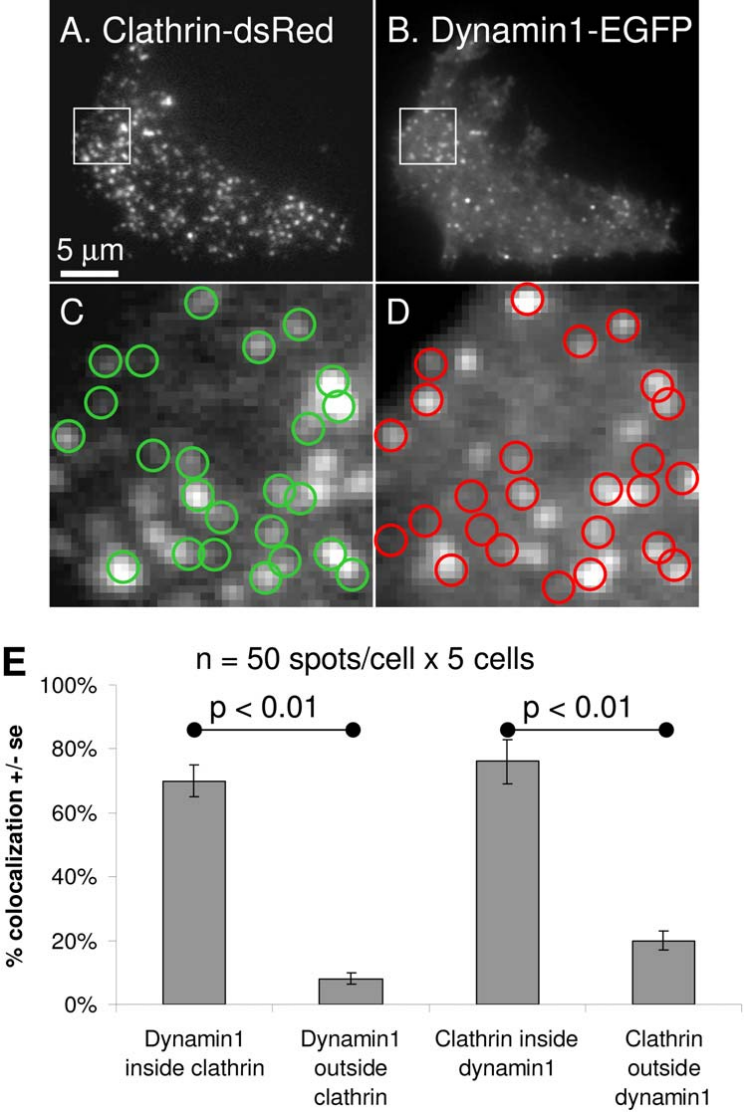
Co-localization of clathrin and dynamin1 in PC12 cells. PC12 cells co-transfected with clathrin-dsRed (A,C) and dynamin1-EGFP (B,D) were imaged by TIR-FM. Regions drawn around clathrin spots (A) were transferred to dynamin1 images (red circles in D). Regions drawn around dynamin1 spots (B) were transferred to clathrin images (green circles in C). 250 spots per group were evaluated to determine if clathrin and dynamin1 co-localize (E). Dynamin1 at sites of clathrin spots is significantly higher than dynamin1 outside clathrin spots. Clathrin corresponding to dynamin1 spots is significantly higher than dynamin1 outside clathrin spots.

**Figure 2:**
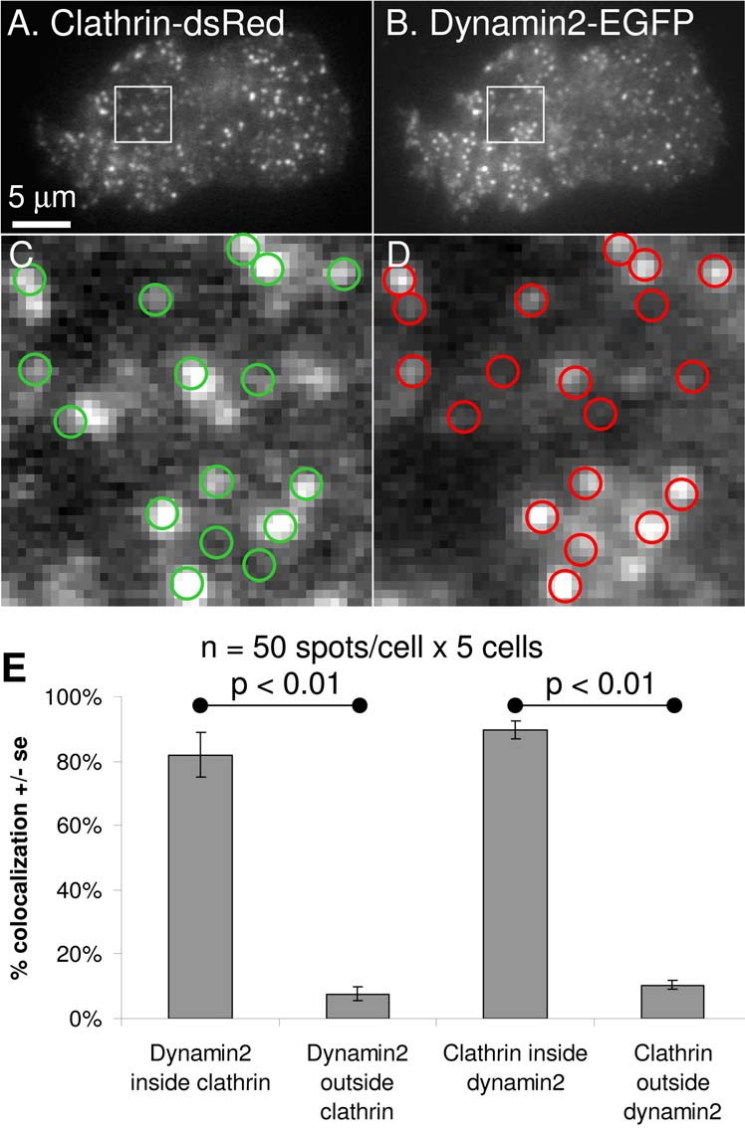
Co-localization of clathrin and dynamin2 in PC12 cells. PC12 cells co-transfected with clathrin-dsRed (A,C) and dynamin2-EGFP (B,D) were imaged by TIR-FM. Regions drawn around clathrin spots (A) were transferred to dynamin2 images (red circles in D). Regions drawn around dynamin2 spots (B) were transferred to clathrin images (green circles in C). 250 spots per group were evaluated to determine if clathrin and dynamin2 co-localise (E). Dynamin2 corresponding to clathrin spots is significantly higher than dynamin1 outside clathrin spots. Clathrin at sites of dynamin2 spots is significantly higher than dynamin1 outside clathrin spots.

Paradigms determined from our previous studies in this area were applied to the current data sets [37]. Spots for co-localization studies (Figures 1 and 2) were identified in single static images by circling with 8 pixel×8 pixel regions in MetaMorph (108 nm per pixel). Only spots which did not overlap with neighbors were circled. Otherwise, identification was random. Spots were recorded as co-localizing if the majority of the spot intensity in the corresponding image from the other channel fit within the confines of the drawn regions.

The criteria for identification and analysis of dynamic spots from image stacks were based upon that for static co-localization studies. However, spots were omitted if any laterally mobile spots, or other separation or coalescence of spots, interfered with identification, tracking or quantification. Furthermore, if the dynamin signal appeared to show multiple flashes, only spots where the first flash was the brightest were analyzed – In order to be able to clearly identify the point at which disappearance began. Regions around spots to be analyzed were drawn to maximize the spot fluorescence and minimize the contribution of local background fluorescence. “Temporal alignment” and normalization of fluorescence intensity data was performed as in our previous studies [38]. Fluorescence from each channel within the period analyzed was normalized relative to the maximum (1) and minimum (0) values for each spot. Spot fluorescence intensity data was aligned, in both channels, so that the maximum intensities of the dynamin signal occurred simultaneously.

## Results

### Co-localization studies of clathrin and dynamin1/dynamin2:

Clathrin and dynamin tagged with fluorescent proteins have been previously employed in numerous live-cell imaging studies without evidence of perturbation of the endocytic system [19], [20], [22], [39], [40]. Although the studies which have employed these fusion proteins have demonstrated proper localization, as well as lack of dominant negative effects, it has not been directly shown that wild type functionality is retained. The use of siRNA to dynamin1 and dynamin2 is problematic since they are expressed as multiple splice variants. However, these types of analyses have yet to be performed in cells which endogenously express both dynamin1 and dynamin2. Forty-eight hours post-transfection numerous PC12 cells were observed under TIR-FM to contain puncta of both clathrin-dsRed and dynamin1-EGFP (Fig. 1A,B). The potential co-localization of clathrin and dynamin1 was quantitatively assessed. Although colocalization is apparent in the representative images presented in Figure 1, no one set of contrast scaling can depict all information relevant to an analysis such as this. Thus multiple cells were evaluated under a wide range of display settings during the quantification of clathrin/dynamin colocalization. Clathrin spots were identified and then these regions were transferred to the dynamin1 images (Fig. 1D, red circles). Similarly, regions drawn at the sites of dynamin1 spots were placed on the clathrin images (Fig. 1C, green circles). Quantification of the clathrin fluorescence at sites of 250 dynamin1 spots revealed significant co-localization (70%, p<0.01), compared to 250 sites where no dynamin1 was evident (8%) (Fig. 1E). Similar findings were observed for the dynamin1 fluorescence present at 250 clathrin spots (p<0.01), relative to 250 sites without clathrin (Fig. 1E).

Very similar observations were obtained when clathrin and dynamin2 were analyzed by the same methods (Fig. 2). However, a few subtle differences were evident suggesting a higher degree of co-localization (82%, p<0.01) between clathrin and dynamin2, relative to clathrin and dynamin1 (70%). The presence of dynamin2 at sites of clathrin was found to be ∼10% higher than the co-localization of dynamin1 with clathrin (p-value = 0.19). Similarly, the number of dynamin 2 puncta that were positive for clathrin was 10% greater than the number of dynamin 1 puncta that contained clathrin (p-value = 0.10). Conversely, clathrin outside of dynamin2 was ∼10% lower than clathrin outside of dynamin1 (p-value = 0.015).

### Dynamic analysis of dynamin increase at sites of clathrin-mediated endocytosis:

**Figure 3:**
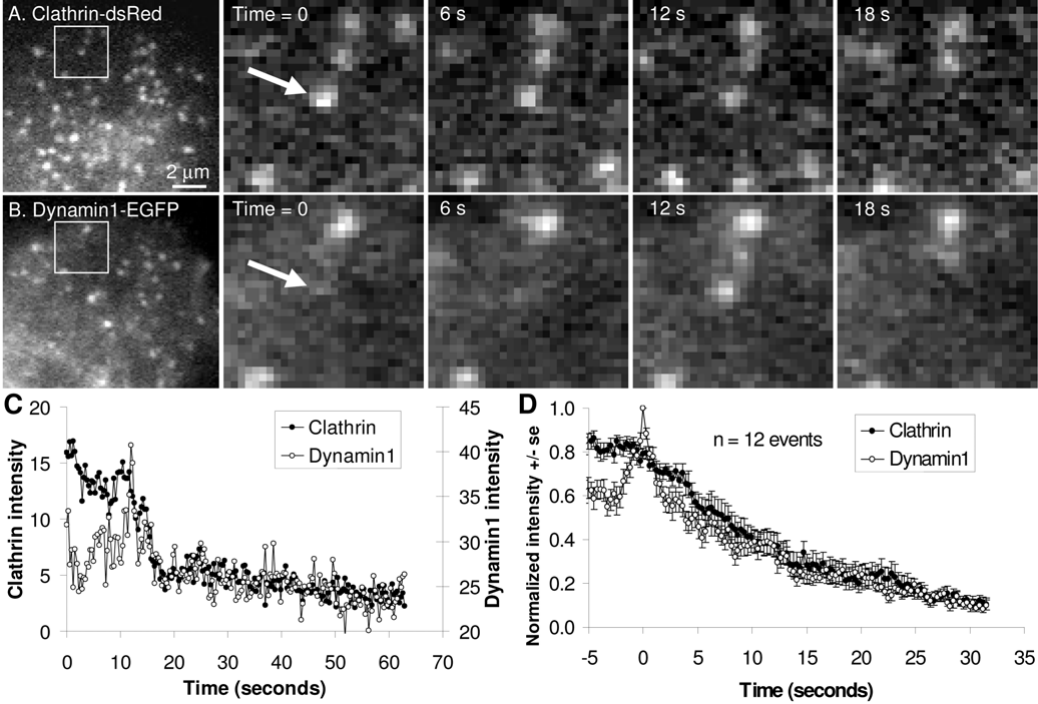
Increase in dynamin1 intensity at sites of clathrin-mediated endocytosis in PC12 cells. PC12 cells co-transfected with clathrin-dsRed (A) and dynamin1-EGFP (B) were imaged by live-cell TIR-FM. Frames with time-stamps represent magnifications of regions identified directly to the left. Quantification of the spot marker by the arrow (C) shows burst of dynamin1 fluorescence just prior to endocytosis. Additionally, clathrin fluorescence remains roughly constant prior to internalization. Quantification of 12 such events is shown is (D).

**Figure 4:**
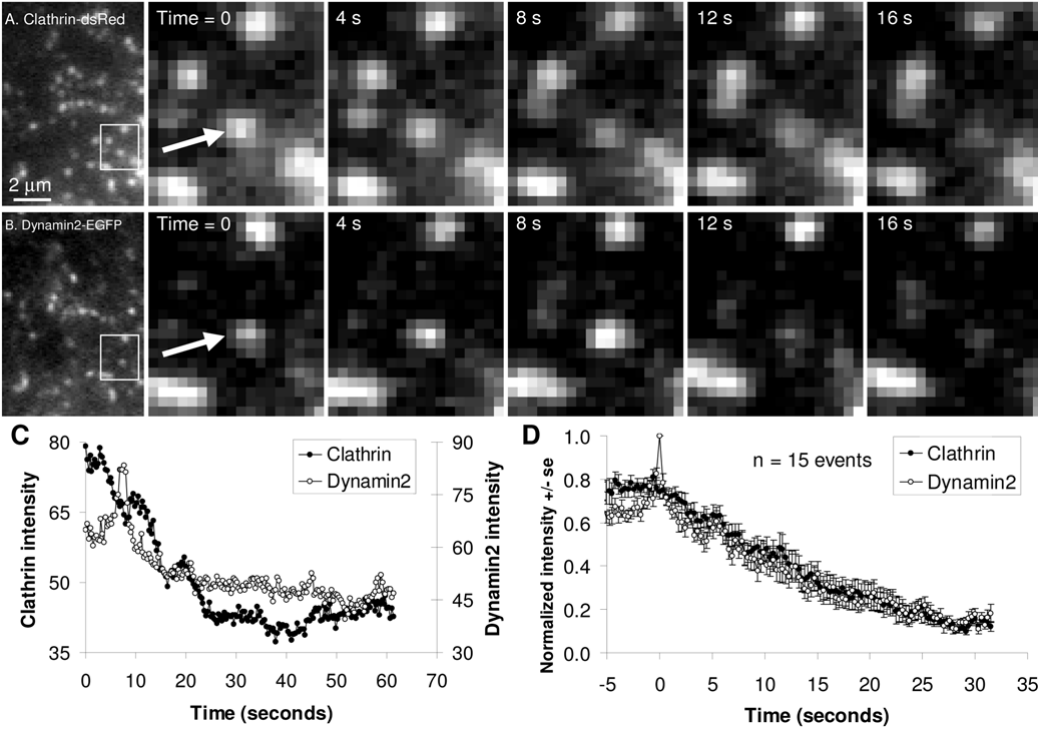
Increase in dynamin2 intensity at sites of clathrin-mediated endocytosis in PC12 cells. PC12 cells co-transfected with clathrin-dsRed (A) and dynamin2-EGFP (B) were imaged by live-cell TIR-FM. Frames with time-stamps represent magnifications of regions identified directly to the left. Quantification of the spot marker by the arrow (C) shows burst of dynamin2 fluorescence just prior to endocytosis. Additionally, clathrin fluorescence remains roughly constant prior to internalization. Quantification of 15 such events is shown is (D).

In order to test whether differences between dynamin1 and dynamin2 during clathrin-mediated endocytosis could be observed, each was imaged simultaneously with clathrin by live-cell TIR-FM in PC12 cells. When individual spots were tracked over time, the clathrin fluorescence in individual puncta was observed to disappear, while the fluorescence at neighboring sites remained unchanged (Figs. 3 and 4, and data not shown). Analysis of over 60 disappearing clathrin spots from each group found no statistically significant difference (p-value = 0.5) between the level of co-localization of dynamin1 at sites of clathrin-mediated endocytosis (75+/−7%) and that of dynamin2, (82+/−3%).

As in previous studies of dynamin1 and clathrin in other cell lines, the dynamin fluorescence increased by 65% over a second, coincident with the onset of clathrin internalization (Fig. 3). This was apparent through visual inspection (Fig. 3A, B), as well as through quantification of individual events (Fig. 3C). Multiple events were aligned by defining the peak of the dynamin1 intensity as t = 0 and the clathrin and dynamin1 intensities normalized between 0 (minimum) and 1 (maximum). An average of multiple events (n = 12), demonstrated that while clathrin fluorescence intensity is nearly constant prior to internalization, there is a rapid burst of dynamin1 at the site of clathrin-mediated endocytosis (Fig. 3D).

The dynamic behavior of dynamin2 during clathrin-mediated endocytosis in PC12 cells was similar to dynamin 1 (Fig. 4). As with dynamin1 (Figure 3), while clathrin fluorescence was approximately constant prior to internalization, dynamin2 increased in intensity by 50%. The similarities between the behaviors of dynamin1 and dynamin2 during clathrin-mediated endocytosis seemed to continue following the rise in fluorescence to its peak value.

### Two-phase decrease in dynamin intensity:

**Figure 5:**
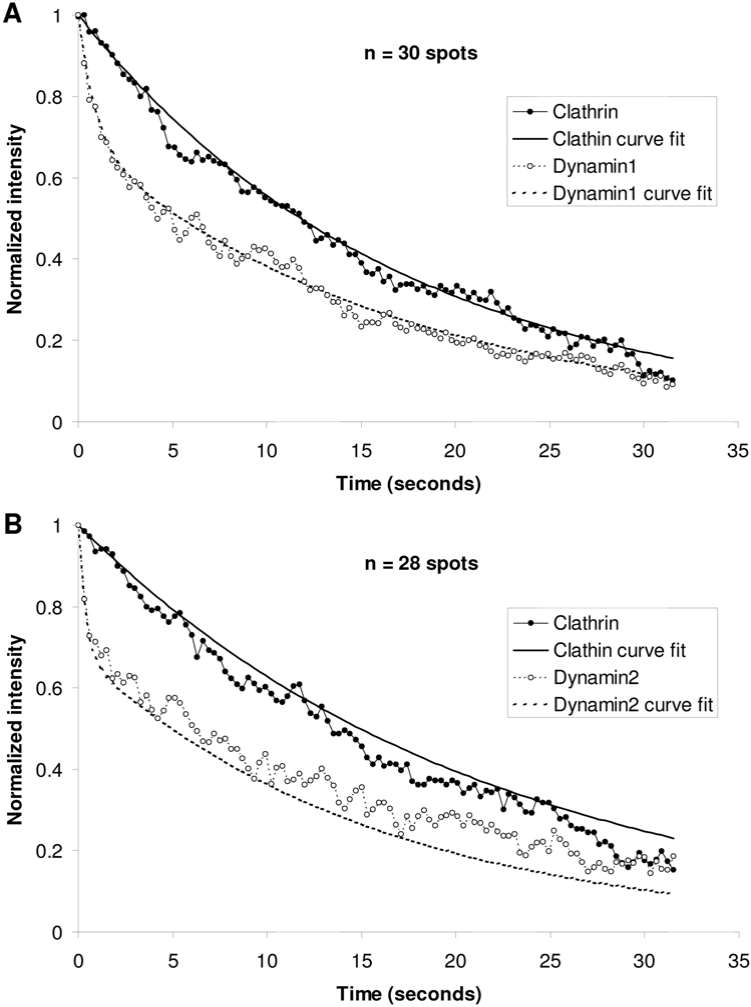
Different phases of dynamin intensity decrease at sites of clathrin-mediated endocytosis in PC12 cells. PC12 cells co-transfected with clathrin-dsRed and dynamin1-EGFP (A) or dynamin2-EGFP (B) were imaged by live-cell TIR-FM. Decreases in dynamin fluorescence were aligned so that the peak was set to time 0, and clathrin and Dynamin fluorescence at time 0 were normalized to 1. (B and C) demonstrate the decreases in clathrin and dynamin1 or dynamin2, at the sites of 30 or 28 disappearing clathrin spots, respectively. In each case, the decrease in clathrin intensity fits a single exponential, while both dynamin1 and dynamin2 fit double exponentials (all r^2^ values >0.98).

The kinetics of decrease of fluorescence intensity values for numerous events were plotted by setting the peak of dynamin intensity at time 0, and normalizing maximum values to 1 (Fig. 5). Analysis of 30 events of dynamin1 fluorescence increase and subsequent decrease (Fig. 5A), and 28 events of dynamin2 (Fig. 5B) at sites of clathrin-mediated endocytosis revealed very similar observations. In both cases two time constants of decrease in the fluorescence of dynamin from its peak value were resolved.

The majority of dynamin1 (70%) decreased with a time constant of 17 seconds, which was the same as the time constant for clathrin disappearance. However, the rest of the dynamin1 decreased with a much faster time constant of 0.89 seconds. The time constant for clathrin decrease at sites of dynamin2 recruitment equals 21.5 seconds. While ∼70% of dynamin2 signal decreased with a time constant of 15.8 seconds, ∼30% decreased with a much faster time constant of 0.36 seconds.

## Discussion

Previously, we, and others, have employed total internal reflection fluorescence microscopy (TIR-FM) to analyze the dynamics of proteins involved in clathrin-mediated endocytosis [41]. Although the analysis of members of the dynamin protein family has been a major focus of this work, to date dynamin1 and clathrin have yet to be imaged in cells that endogenously express dynamin1 [18]–[20], [22], [42], [43]. Furthermore, although model systems exist in cellular neuroscience for imaging exocytic phenomena (e.g. PC12 cells and bovine chromaffin cells), a cellular model competent for the analysis of individual events of clathrin-mediated endocytosis does not yet exist [31], [44]–[46]. Thus, the present study focuses on utilization of PC12 cells for imaging clathrin and dynamin during endocytosis.

Our results demonstrate that both dynamin1 and dynamin2 significantly co-localise with clathrin in PC12 cells. The observation that ∼70% of the clathrin puncta are positive for dynamin1 and ∼80% of the clathrin puncta are positive for dynamin2 suggests that a substantial percentage of the clathrin puncta are positive for both dynamins. Additionally, the clathrin puncta that were disappearing, and thus endocytosing, were equally labeled with dynamin1 and dynamin2. These results do not provide evidence for a functional difference between dynamin1 and dynamin2 in these cells. Thus, for some endocytic events they may have redundant functions and, at least in these non-stimulated PC-12 cells, under the growth conditions used, the minor differences discerned in individual static images may not reflect a functional disparity.

Prior to internalization, the dynamin was observed to increase in parallel with clathrin. However, just before the clathrin puncta internalized the fluorescence intensity of either dynamin1 or dynamin2 increased over a second by 50–65%. As the complete duration of dynamin ‘burst’ only lasted ∼2 seconds, this demonstrates the need for rapid sampling when performing live-cell imaging studies of the dynamics of endocytosis. This increase in the fluorescence could result from the recruitment of additional dynamin. Alternatively, it may not reflect an increase in the number of dynamin molecules, but may reflect a physical movement of dynamin molecules to the neck of the vesicle. This would move them closer to the cover slip and increase their fluorescence excitation. If we assume that the evanescent field decays with a space constant of 70 nm and the vesicle has a radius of 35 nm then the fluorescence should increase 58%. The larger the radius of the vesicle, the greater the predicted increase of fluorescence (see table). This calculation assumes that that dynamin initially is evenly distributed over the membrane, and then 100% of the dynamin moves to the interface between the vesicle and the plasma membrane. If less than all of the dynamin moves, the predicted increase would be less. However, the calculation exists that the increase could be sufficient to account for the observed increase.

**Table:** 

Radius (nm)	35	40	45	50	55
Percentage increase	58%	68%	78%	88%	98%

While the behaviors of dynamin1 during clathrin mediated endocytosis in PC12 cells were similar to those observed in other cells lines that lacked dynamin 1, the dynamics of dynamin2 were different than seen in some previous studies [22], [47]. In our previous analysis of clathrin and dynamin2 in MDCK cells, the kinetics for these proteins were similar. They were marked by a gradual rise in fluorescence of both clathrin and dynamin2 prior to the endocytosis event [48]. However, in PC12 cells the behaviors observed for dynamin2 were nearly identical to those of dynamin1 (Fig. 3), a marked increase prior to internalization. Furthermore, in contrast with some other studies the clathrin fluorescence was constant prior to internalization, suggesting possible differences in the modes of endocytosis between PC12 cells and these other models [18], [22], [49]. It is possible that during fission of an endocytic vesicle from the membrane, only a fixed amount of dynamin is recruited to the vesicular neck. If in our previous studies, the fluorescent dynamin was over-expressed to a high enough degree, we might not have detected the increased fluorescence of dynamin that was recruited, or migrated to the neck..

Previously we have observed within each endocytic puncta, other endocytic markers such as epsin and endocytic cargo such as transferrin leaves the evanescent field with a time course that is indistinguishable from clathrin [50], [51]. Our observations that 30% of the dynamin 1 or 2 rapidly dissociates from the clathrin during endocytic internalization is consistent with models that posit that a key role of dynamin is during vesicle fission form the membrane. The “fast” phase of decrease could reflect diffusion of dynamin monomers following loss of self association after [52]. However, the observation that ∼70% of dynamin internalizes does so with clathrin, suggests that much of the dynamin remains associated with the nascent vesicle throughout the internalization process. Thus, the presence of two distinct kinetics of dynamin disappearance from sites of endocytosis might provide evidence in favor of a dual function role. Future studies could demonstrate that dynamin functions as both a pinchase (“fast” pool) and as a regulatory GTPase (“slow” pool).
